# Supplementary figures and images for: Viral Biomarker Detection and Validation Using MALDI Mass Spectrometry Imaging (MSI)
Source: Proteomes. 2022 Sep 13;10(3):33. doi: 10.3390/proteomes10030033 (PMC9506211; doi:10.3390/proteomes10030033)

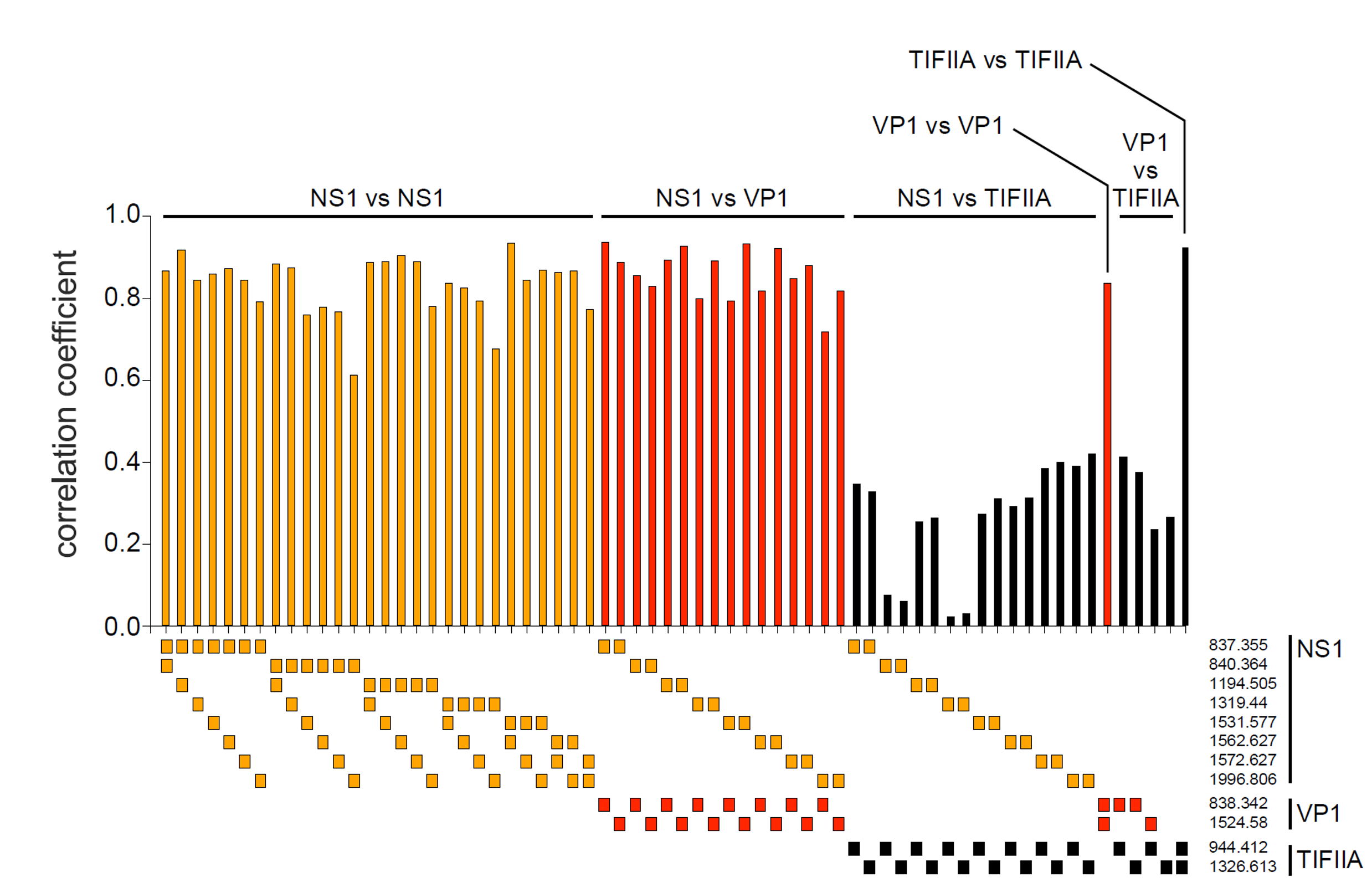

Supplement: Supplementary file 1 [file proteomes-10-00033-s001.zip › Supplementary Figure S1.tif]
